# Hypoxia‐induced FOXO4/LDHA axis modulates gastric cancer cell glycolysis and progression

**DOI:** 10.1002/ctm2.279

**Published:** 2021-01-15

**Authors:** Xiao‐Hong Wang, Zhong‐Hua Jiang, Hong‐Mei Yang, Yu Zhang, Li‐Hua Xu

**Affiliations:** ^1^ Department of Gastroenterology The Second Affiliated Hospital of Xuzhou Medical University Xuzhou Jiangsu China; ^2^ Department of Gastroenterology The Yancheng Clinical College of Xuzhou Medical University Yancheng Jiangsu China; ^3^ Department of Gastroenterology The First People's Hospital of Yancheng Yancheng Jiangsu China; ^4^ Department of Gastroenterology The Sixth People's Hospital of Nantong Nantong Jiangsu China

**Keywords:** Forkhead box O4, Gastric cancer, Glycolysis, Hypoxia‐inducible factor‐1α, Lactate dehydrogenase A

## Abstract

**Background and aim:**

We previously identified forkhead box (FOX) O4 mRNA as a predictor in gastric cancer (GC). However, the underlying mechanism has yet to be elucidated. We aimed to illustrate the mechanism by which FOXO4 regulated glycolysis under hypoxia in GC.

**Methods:**

FOXO4 protein expression was investigated by immunohistochemical staining of 252 GC and their normal adjacent tissues. We restored or silenced FOXO4 expression in GC cell lines to explore the underlying mechanisms.

**Results:**

FOXO4 was downregulated in GC. Loss of FOXO4 expression was validated in univariate and multivariate survival analysis as an independent prognostic predictor for overall survival (*P *< 0.05) and disease‐free survival (*P*<0.05). Restored FOXO4 expression significantly impaired the glycolysis rate in GC cells, while silencing FOXO4 expression enhanced glycolysis rate. FOXO4 expression was inversely associated with maximum standardized uptake value in mice models and patient samples. Mechanistically, FOXO4 bound to the glycolytic enzyme lactate dehydrogenase (LDH)A promoter and inactivated its activity in a dose‐dependent manner (P < 0.05). Finally, we determined that FOXO4 was a transcriptional target of hypoxia‐inducible factor (HIF) ‐1α, which is central in response to hypoxia.

**Conclusions:**

Our data suggested that FOXO4 plays a key role in the regulation of glycolysis in GC, and disrupting the HIF‐1α‐FOXO4‐LDHA axis might be a promising therapeutic strategy for GC.

## Graphical abstract

FOXO4 was frequently downregulated in gastric cancer and may serve as a biomarker for prognosis. FOXO4 expression was associated with glycolysis rate both in vitro and in vivo. Mechanistically, FOXO4 impaired glycolysis by transcriptional inhibiting glycolytic enzyme LDHA, and FOXO4 itself was directly by HIF‐1α.

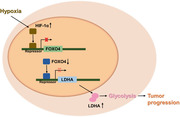

**Abbreviations**
DFS: disease‐free survival
FOX: Forkhead box
GC: gastric cancer
IHC: immunohistochemical
OS: overall survival
PET/CT: positron emission tomography/computed tomography.
TCGA: The Cancer Genome Atlas

## 1 INTRODUCTION

Gastric cancer (GC) is one of the most common malignant tumors in the world with the highest incidence in China and Japan.^1^ Although significant advancement has been made in surgical techniques and chemotherapy, the prognosis of GC is still poor, mainly because of tumor progression and recurrence. Therefore, it is necessary to understand the biological and genetic characteristics of GC.

One of the fundamental characteristics of tumors is uncontrolled growth. Proliferating cancer cells require more material and energy supplies to maintain their proliferative activity. However, solid tumors always under severely hypoxic conditions with limited oxygen and nutrient supply. To overcome such unfavorable conditions, tumor cells have to remodel their metabolic patterns to adapt to the microenvironment.^2^ This is now regarded as a hallmark of cancer, and has been given more attention than before.^3^ One type of such metabolic reprogramming is called the Warburg effect.^4^ Compared with well‐differentiated normal cells relying on the oxidation of pyruvate to produce energy for physiological function, rapid‐proliferating tumor cells rely on glycolysis to provide energy, even with sufficient oxygen. Although the efficiency of ATP produced by glycolysis is low, it rapidly provides an energy supply for tumor cells and macromolecular substances for molecular synthesis, and forms an acidic environment to facilitate tumor cell metastasis. Therefore, identifying the key mediators of the glycolytic pathway will provide effective strategies in the diagnosis and treatment of GC.

Forkhead box (FOX) transcription factors are classic transcriptional regulators with a winged helix domain to bind to genomic DNA to regulate a variety of biological processes.^5^ Previously, we have shown that FOXO4 mRNA level can be used to predict survival of GC from both The Cancer Genome Atlas(TCGA) and our own database.^6^ However, the underlying mechanisms of FOXO4 in GC progression and metastasis remain elusive. Hence, we first investigated FOXO4 protein expression in a series of GC samples from patients who underwent radical gastrectomy. We then showed FOXO4 as a tumor suppressor through both *in vitro* and *in vivo* studies in GC. Mechanistically, FOXO4 inhibited the glycolysis rate by directly inhibiting glycolytic enzyme lactate dehydrogenase (LDH)A, and FOXO4 was a downstream target of hypoxia‐inducible factor (HIF)‐1α, a well‐established regulator in GC glycolysis.

## 2 PATIENTS AND METHODS

### 2.1 Patient specimens

The patients cohort included 252 cases of GC as described previously.^7^, ^8^ The research was approved by the ethics committee of The First People's Hospital of Yancheng.

### 2.2 Immunohistochemical (IHC) staining

The IHC study was performed as described previously.^7^, ^8^ The rabbit polyclonal antibody against FOXO4 (ab63254 , 1; 250; Abcam, Cambridge, MA, USA) was used as the primary antibody. PBS was used as a negative control. A semi‐quantitative immunoreactivity score (IS) which was calculated by multiply the percentage of stained cells and staining intensity was used.^9^ High FOXO4 expression was defined as IS≥4.^10^

### 2.3 Stable transfection of GC cells

Human GC cell lines, MGC‐803, AGS, SGC7901, and MKN28, were originally purchased from the Chinese Academy of Sciences. Short hairpin RNA (shRNA) construct against FOXO4 was 5′‐AGGCTTTGTAGCAAGA‐3′. FOXO4 stably overexpression or knockdown cells were isolated using puromycin selection.

### 2.4 Real‐time polymerase chain reaction

Total RNA retraction and cDNA preparation were performed as previously described.^8^ The expression status of the different genes was detected by real‐time PCR using an ABI 7900HT PCR system. Primers for RT‐PCR study are listed in Table S1.

### 2.5 Western blot analysis

Western blotting was performed as described previously.^7^, ^8^ The primary antibodies included FOXO4 (ab63254 , 1: 1000; Abcam, Cambridge USA), HIF‐1α( 209601‐1‐AP , 1:1000, Proteintech, Wuhan, China), LDHA ( 19987‐1‐AP , 1:500; Proteintech, Wuhan, China) andβ‐actin (14395‐1‐AP,1:1000; Proteintech, Wuhan, China). β‐actin was used as an endogenous control.

### 2.6 Glycolysis analysis

GC cells glycolysis rate was assessed using Glucose Uptake Colorimetric Assay Kit and Lactate Colorimetric Assay Kit (Biovision, Milpitas, CA, USA) according to the standard protocol. Cellular mitochondrial function and glycolytic capacity were measured as previous described.^11^

### 2.7 Dual luciferase assay

A 2.0‐kb LDHA or 0.8‐kb FOXO4 promoter sequence was cloned into pGL3‐Basic Luciferase Reporter Vectors. Luciferase activities were quantified with a Dual‐Luciferase Reporter Assay System (Promega).

### 2.8 Chromatin immunoprecipitation (ChIP) assay

Chromatin Immunoprecipitation Assay Kit (EMD Millipore) was used for ChIP assay. The primers for HIF‐1α regulation of FOXO4 were as follows. Site 1#: 5′‐ AATCAGAGGAAGATTTAACC ‐3′ (Forward) and 5′‐ CAGTGGTGTGATCTTGGCTC ‐3′ (Reverse). Site 2#:5′‐ TGTCACCCAGGCTGGAGCAC ‐3′ (Forward) and 5′‐ CAAGACCTATTTATAACTGC ‐3′ (Reverse). Site 3#: 5′‐ TGAGGAGGAACACCACCGTG ‐3′ (Forward) and 5′‐ GAGTCGTGTAGGTGACCAGA ‐3′ (Reverse). The primers for FOXO4 regulation LDHA were as follows. Site 1#: 5′‐ AGGTCTGAAGTCTGAATCCCAG ‐3′ (Forward) and 5′‐ CGCGGTTTATTAACCCCAA ‐3′ (Reverse). Site 2#:5′‐ CCCCCTGCCAGGCTAGAAAC ‐3′ (Forward) and 5′‐ AATGAATGCCCCGAAGCAGA ‐3′ (Reverse). Site 3#: 5′‐ CCGGGGCGGGTTCTTGAAA ‐3′ (Forward) and 5′‐ AAGGGAGTTCCTGCGGACAC ‐3′ (Reverse). Site 4#: 5′‐ CGCGCCCAGCTCAGAGTGC ‐3′ (Forward) and 5′‐ ACAAGCTGAGGCTTTTTTGGC ‐3′ (Reverse).

### 2.9 Mouse models and PET/CT study

Gastric cancer cells (5×10^6^ per mouse) were inoculated subcutaneously on both forelimbs of nude mice. All mice were sacrificed 23 days after injection, and their tumors were removed and weighed. All mice were fasted for 8 h before giving 6 μCi 18F‐FDG per gram of body weight for positron emission tomography (PET)/computed tomography (CT) scan before sacrificed.^12^ The study was complied with the animal care guidelines at The First People's Hospital of Yancheng.

### 2.10 Statistical analysis

Statistical tests were conducted with SPSS 21.0 software (Chicago, IL, USA). The relationship between clinical parameters and FOXO4 expression was determined by χ^2^ test. The overall survival (OS) and disease‐free survival (DFS) were estimated with the Kaplan‐Meier method and analyzed with the log‐rank test. All in vitro study were repeated in triplicate. *P* < 0.05 was considered statistically significant.

## 3 RESULTS

### 3.1 FOXO4 downregulation indicated poor prognosis in GC

**FIGURE 1:**
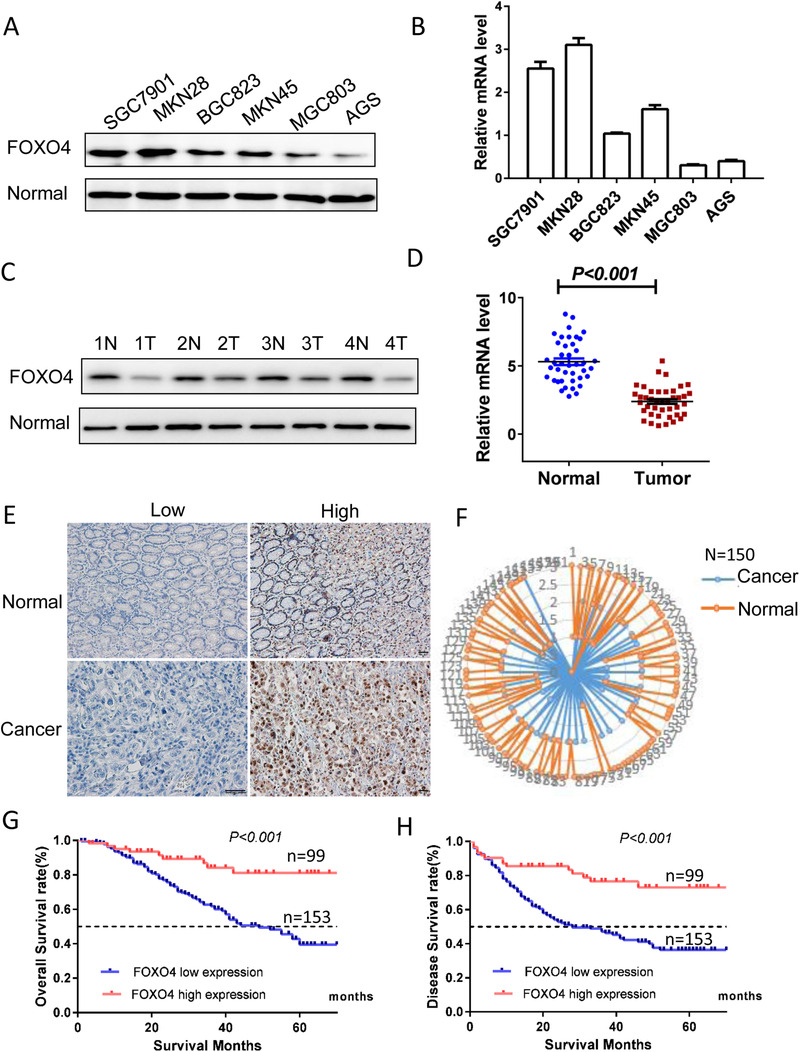
FOXO4 downregulation was associated with poor prognosis in GC. (A, B) FOXO4 expression in six GC cells determined by Western blot(A) and qPCR(B). (C) FOXO4 expression in GC tissues determined by Western blot (C) and qPCR(D). (E) Representative IHC images of FOXO4 expression in GC and normal control tissues. (F) FOXO4 was downregulated in GC specimens compared with the surrounding benign tissues by IHC study(n = 150, *P* < 0.05). (G, H) Kaplan‐Meier analysis of the correlation between FOXO4 expression and overall survival (HR: 0.44, 95%CI: 0.29‐0.68, *P *< 0.001) (G) or disease‐free survival (HR: 0.43, 95%CI: 0.29‐0.64, *P *< 0.001) (H) in GC. Log‐rank tests were used to determine statistical significance(n = 252)

**FIGURE 2:**
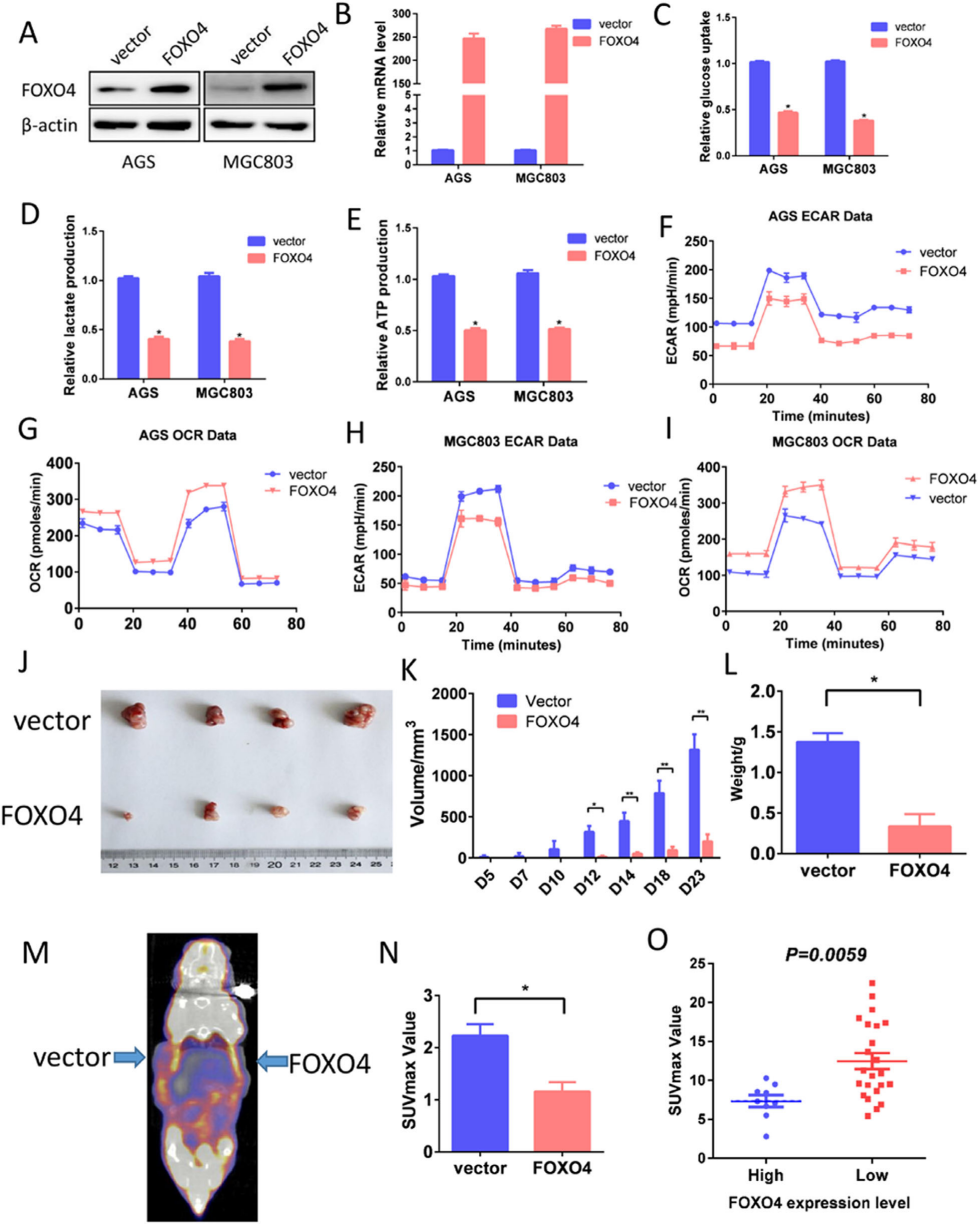
Loss of FOXO4 contributed to GC glucose reprogramming. (A, B) Ectopic expression of FOXO4 in GC cells was validated by western blotting (A) and qPCR (B). (C‐E) Forced expression of FOXO4 impaired glycolysis as determined by decreased glucose consumption (C), lactate production (D), and ATP production (E) in GC cells. (F‐I) Impact of FOXO4 on glycolysis rate was assessed using Seahorse Energy Flux system through examination of ECAR and OCAR, which reflected the glycolytic and mitochondrial respiration, respectively. (J‐L) AGS‐vector/AGS‐FOXO4 cells (1×10^6^) were injected in to right or left forelimbs of nude mice. (J) Gross tumors in the mice were showed. Tumor volumes (K) and tumor weight (L) were measured on the indicated days. (M) Representative photograph of PET/CT scans from AGS‐vector and AGS‐FOXO4 cells formed tumors. (N) The SUVmax was lower in the AGS‐FOXO4 group than in the control group (*P *< 0.05). (O) FOXO4 expression was negatively correlated with SUVmax levels in GC patients *(P *< 0.05)

To assess the role of FOXO4 in GC, we first analyzed FOXO4 expression in six GC cells by Western blot and RT‐PCT and found the expression levels of FOXO4 seem associated with malignancy of GC cells (Figure 1A, B). Then, we evaluated FOXO4 expression in GC tissues by Western blot (Figure 2C) and RT‐PCT(Figure 2D), the results demonstrated that FOXO4 was significantly lower in GC tissue when compared with their paired normal gastric mucosa.

To further determine the clinical significance of FOXO4 protein expression in GC, we analyzed FOXO4 protein levels in 150 pairs of human GC and their corresponding noncancerous adjacent gastric tissues by IHC staining. Representative images of GC and gastric tissues IHC staining are shown in Figure 1E. Compared with corresponding nontumorous tissues, FOXO4 was significantly downregulated in GC tissues (24.67% vs 84.67%, *P *< .001) (Figure 1F).

Moreover, FOXO4 protein expression was investigated in another 252 GC patients. FOXO4 expression was negatively associated with tumor diameter and the presence of lymph node metastasis (Table.1). Kaplan–Meier survival curves revealed that OS in the high and low FOXO4 expression subgroups was 80.9% and 42.4%, respectively. Log‐rank analysis demonstrated that the differences were significant (*P *< .001) (Figure 1G). Similarly, patients with low FOXO4 expression have a significantly worse 5‐year DFS than that of high expression(*P *< .001) (Figure 1H). Importantly, downexpression of FOXO4 was significantly correlated with shorter OS [hazard ratio (HR) : 0.393, 95% confidence interval (CI): 0.191‐0.808, *P *= .011] (Table 2) and DFS (HR: 0.409, 95%CI: 0.229‐0.730, *P *= .003) in multivariate Cox regression analysis (Tables 2 and 3). Taken together, our data show that FOXO4 is downregulated in GC and related to clinical severity and prognosis.

**TABLE 1:** Association between FOXO4 expression and clinicpathological factors in gastric cancers

		FOXO4 expression		
Characteristics	Total	Low expression	High expression	*P* value
		N (%)	N (%)	
Gender				.564
Male	134	103(76.9)	31(23.1)	
Female	118	87(73.7)	31(26.3)	
Age				.344
≥60	121	88(72.7)	33(27.3)	
<60	131	102(77.9)	29(22.1)	
Primary site				.166
Antrum/Distal	95	74(77.9)	21(22.1)	
Cardia/Proximal	86	60(69.8)	26(30.2)	
Fundus/Body	48	35(72.9)	13(27.1)	
Gastroesophageal Junction	21	5(91.3)	2(8.7)	
Diameter(cm)				**.001**
<4	160	110(68.8)	50(31.3)	
≥4	92	80(87.0)	12(13.0)	
Histologic grade				.311
G1/G2	108	78(72.2)	30(27.8)	
G3	144	112(77.8)	32(22.2)	
Histological type				.868
Adenocarcinoma mucinous adenocarcinoma/	221	167(75.6)	54(24.4)	
signet ring cell cancer	31	23(74.2)	8(25.8)	
T stage				.066
T1/2	40	25(62.5)	15(37.5)	
T3	111	83(74.8)	28(25.2)	
T4	101	82 (81.2)	19(18.8)	
N stage				**<.001**
N0	69	36(52.2)	33(47.8)	
N1	52	41(78.8)	11(21.2)	
N2	58	47(81.0)	11(19.0)	
N3	73	66(90.4)	7(9.6)	
Lymphovascular invasion				.184
Negative	196	144(73.5)	52(26.5)	
Positive	56	46(82.1)	10(17.9)	
Perineural invasion				.249
Negative	185	136(73.5)	49(26.5)	
Positive	67	54(80.6)	13(19.4)	

**TABLE 2:** Univariate and multivariate Cox proportional hazards analysis of FOXO4 gene expression and overall survival for patients with gastric cancer

**Factor**	**Univariate analysis**		**Multivariate analysis**	
	**HR (95% CI)**	**P**	**HR (95% CI)**	***P***
Gender	0.780 (0.514‐1.183)	0.242		
Age	1.262(0.832‐1.915)	0.273		
Diameter	1.254(0.810‐1.941)	0.309		
T stage	2.029(1.479‐2.782)	**<0.001**	1.255(0.815‐1.932)	.303
N stage	1.652(1.365‐2.000)	**<0.000**	1.356(1.050‐1.750)	**.019**
Grade	1.694(1.094‐2.625)	**0.018**	1.169(0.738‐1.851)	.506
Lymphovascular invasion	2.193(1.404‐3.426)	**0.001**	1.818(1.145‐2.889)	**.011**
Perineural invasion	1.835(1.193‐2.822)	**0.006**	1.620(1.040‐2.523)	**.033**
Tumor location	1.149(0.957‐1.379)	0.137		
FOXO4	0.284(0.143‐0.567)	**<0.001**	0.393(0.191‐0.808)	**.011**

Abbreviations: CI, confidence interval; HR, hazard ratio.

Bold type indicates statistical significance.

**TABLE 3:** Univariate and multivariate Cox proportional hazards analysis of FOXO4 gene expression and disease free survival for patients with gastric cancer

**Factor**	**Univariate analysis**		**Multivariate analysis**	
	**HR (95% CI)**	***P***	**HR (95% CI)**	***P***
Gender	0.949(0.662‐1.361)	.775		
Age	1.132(0.789‐1.624)	.500		
Diameter	1.187(0.814‐1.729)	.373		
T category	2.068(1.568‐2.728)	**<.001**	1.475(1.037‐2.099)	**.031**
N stage	1.477(1.260‐1.732)	**<.001**	1.156(0.944‐1.414)	**.160**
Grade	1.917(1.302‐2.822)	**.001**	1.343(0.899‐2.005)	.150
Lymphovascular invasion	2.205(1.497‐3.248)	**<.001**	1.984(1.331‐2.958)	**.001**
Perineural invasion	1.503(1.022‐2.211)	**.038**	1.241(0.835‐1.845)	.285
Tumor location	1.069(0.910‐1.257)	.416		
FOXO4	0.325(0.186‐0.529)	**<.001**	0.409 (0.229‐0.730)	**.003**

Abbreviations: CI, confidence interval; HR, hazard ratio.

Bold type indicates statistical significance.

### 3.2 Loss of FOXO4 contributes to GC glucose reprogramming

Reduced FOXO4 levels were associated with advanced N stage and large tumor size in GC (Table 1). Glycolysis is one of the fundamental characteristics of cancer. In order to illustrate the effects of FOXO4 inactivation on glycolysis in GC cells, we assessed the relationship between FOXO4 expression and glycolysis rate *in vitro*. We stably ectopic expressed FOXO4 in AGS and MGC803 cells, and the efficiency was illustrate by Western blotting (Figure 2A) and RT‐PCR (Figure 2B). Ectopic expression of FOXO4 led to a significant decrease in glucose consumption and lactate and ATP production (Fig, 2C, D). Conversely, silencing FOXO4 expression significantly enhanced GC cell glycolysis rate (Figure S1). Then, Seahorse XF Extracellular Flux Analyzers were used to determine the impact of FOXO4 on GC aerobic glycolysis which was shown through ECAR and OCR. ECAR is an indicator of glycolytic activity by reflecting the acidity in the microenvironment. OCR is an indicator of oxygen consumption caused by mitochondrial respiration.^11^ ECAR decreased significantly, while OCR value increased significantly after overexpression FOXO4 (Figure 2F‐I).

In the nude mice model, we found that overexpression of FOXO4 significantly reduced the tumor‐forming capacity of AGS cells (*P *< 0.05) (Figure 2J‐L). Aerobic glycolysis is the theoretical basis of clinical PET/ CT examination. All above‐mentioned nude mice underwent PET/CT study before being sacrificed. The results indicated that overexpression of FOXO4 significantly reduced the maximum standard uptake value (SUVmax) value when compared with control mice (Figure 2 M,N). More importantly, FOXO4 expression was inversely correlated with SUVmax level in patients who underwent PET/CT scan after the initial diagnosis of GC (Figure 2O).

### 3.3 LDHA is a transcriptional target of FOXO4 in GC

**FIGURE 3:**
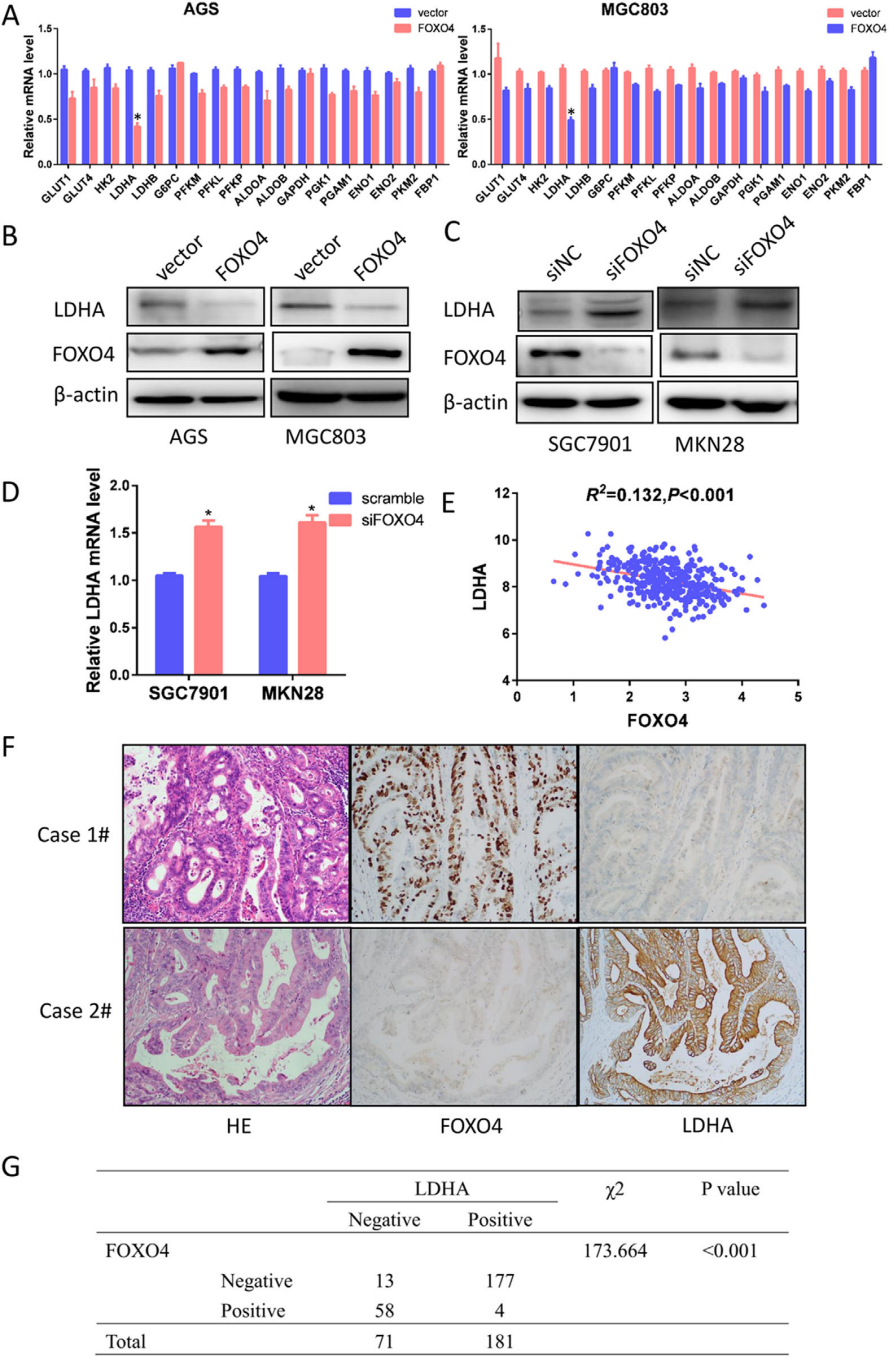
FOXO4 affected glycolytic enzyme LDHA expression in GC. (A)The RT‐PCR analysis demonstrated the mRNA change of glycolytic enzymes after forced FOXO4 expression with the most downregulation was LDHA. (B)The change in protein level of LDHA was validated by western blotting. (C, D) Silencing FOXO4 expression upregulated LDHA at both protein(C) and transcriptional levels(D) in SGC7901 and MKN28 cells. (E) FOXO4 was inversely associated with LDHA expression in the TCGA database (*P *< 0.001). (F, G) Immunohistochemistry demonstrated that FOXO4 expression was negatively correlated with LDHA expression in GC samples (*P *< 0.05). *P < 0.05

To explore further the mechanism by which FOXO4 inhibited glycolysis in GC, we investigated the effect of ectopic FOXO4 expression on glycolytic enzymes. RT‐PCR analysis indicated that LDHA mRNA levels decreased significantly after ectopic FOXO4 expression (Figure 3A). Western blotting analysis verified the same change in protein level (Figure 3B). Consistently, silencing FOXO4 expression had an opposite effect on LDHA expression in SGC7901 and MKN28 cells (Figure 3C, D). In the TCGA database, FOXO4 expression was also inversely associated with LDHA expression at the transcriptional levels (*P *< 0.001) (Figure 3E). Consistent with these findings, high FOXO4 expression was accompanied with low LDHA expression in the validation cohort consisting of 181 GC samples (*P *< .001; Figure 3F,G). Therefore, LDHA may be a transcriptional target of FOXO4.

**FIGURE 4:**
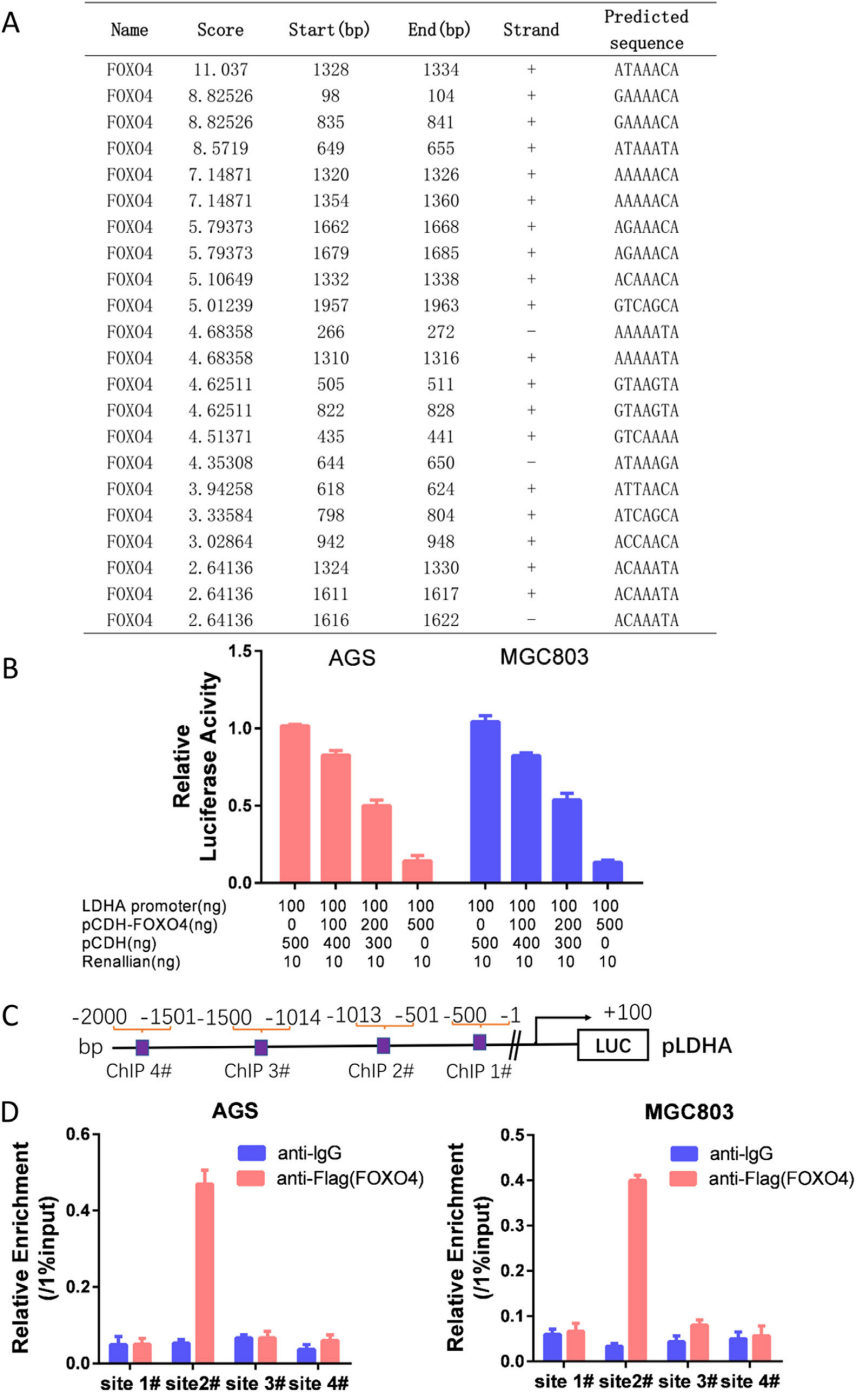
LDHA was a transcriptional target of FOXO4 in GC. (A) By using the JASPAR CORE database, we identified a series of putative binding sites in LDHA promoter for FOXO4 with calculated z‐score from 11.037 to 2.641. (B) Luciferase assays demonstrated that FOXO4 significantly decreased the luciferase activity of LDHA in both AGS and MGC803 cells in a dose‐dependent manner. (C) Schematic of ChIP analysis in LDHA promoter. (D) Chromatin immunoprecipitation analysis demonstrated that FOXO4 was directly bound to the region from ‐501 to ‐1013 bp of the LDHA promoter (sites 2#)

FOXO4 is a classic transcription factor. By using the JASPAR CORE database,^13^ we identified in LDHA promoter a series of putative binding sites for the transcription factor FOXO4, with calculated *z*‐score of 11.037‐2.641(Figure 4A). Luciferase assays demonstrated that FOXO4 could decrease the luciferase activity of LDHA markedly in both AGS and MGC803 cells in a dose‐dependent manner (Figure 4B). As there were multi potential bound sites of FOXO4 in LDHA promoter, we then designed ChIP analysis as demonstrated in Figure 4C, ChIP analysis indicated that FOXO4 could directly bind to the region from ‐501 to ‐1013 bp of the LDHA promoter (sites 2#) (Figure 4D).

### 3.4 Altered LDHA expression ablates FOXO4 effect on glycolysis

**FIGURE 5:**
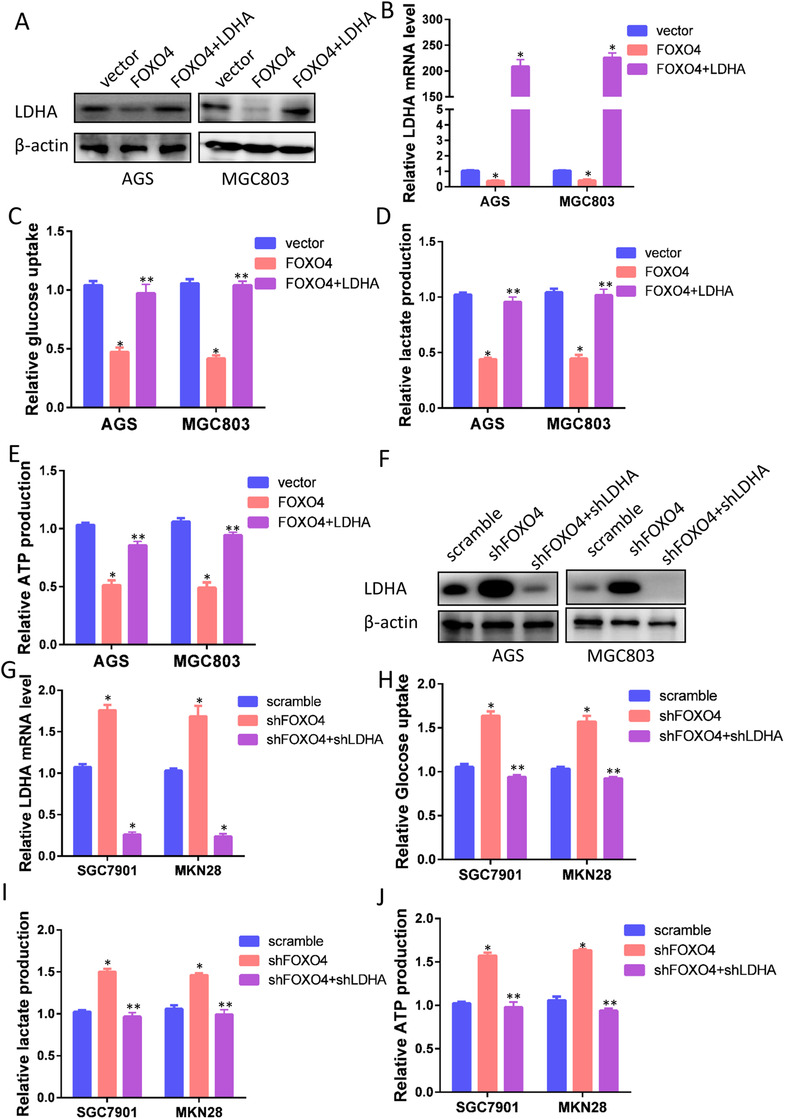
Restored LDHA expression ablated FOXO4 inhibition of glycolysis. (A, B) Overexpression of LDHA in AGS‐FOXO4 and MGC803‐FOXO4 cells was determined by western blotting (A) and RT‐PCR (B). (C‐E) Restored LDHA expression in cells with ectopic FOXO4 expression reversed the effect of FOXO4 on glycolysis. (F, G) Knockdown effect of LDHA in SGC7901‐shFOXO4 and MKN28‐shFOXO4 cells was determined by western blotting (F) and RT‐PCR (G). (H‐J) Knockdown of LDHA expression in FOXO4 knockdown cells reversed the enhancing effect of silencing FOXO4 on glycolysis. *P < 0.05.**P > 0.05

To confirm LDHA as a transcriptional target of FOXO4, we restored LDHA expression in cells with stable overexpression of FOXO4. As demonstrated in Figure 5A, B, we successfully restored LDHA expression in both AGS‐FOXO4 and MGC803‐FOXO4 cells. Glycolysis analysis indicated that restored LDHA expression in cells with ectopic expression of FOXO4 reversed the effect of FOXO4 on glycolysis (Figure 5C‐E). Similarly, we silenced LDHA expression in FOXO4 knockdown cells as demonstrated in Figure 5F, G. As anticipated, silencing LDHA expression in FOXO4 knockdown cells impaired its enhancement of glycolysis in GC cells (Figure 5H‐J). Collectively, these results suggest that FOXO4 attenuates GC glycolysis by targeting LDHA.

### 3.5 FOXO4 is directly regulated by the transcription factor HIF‐1α

**FIGURE 6:**
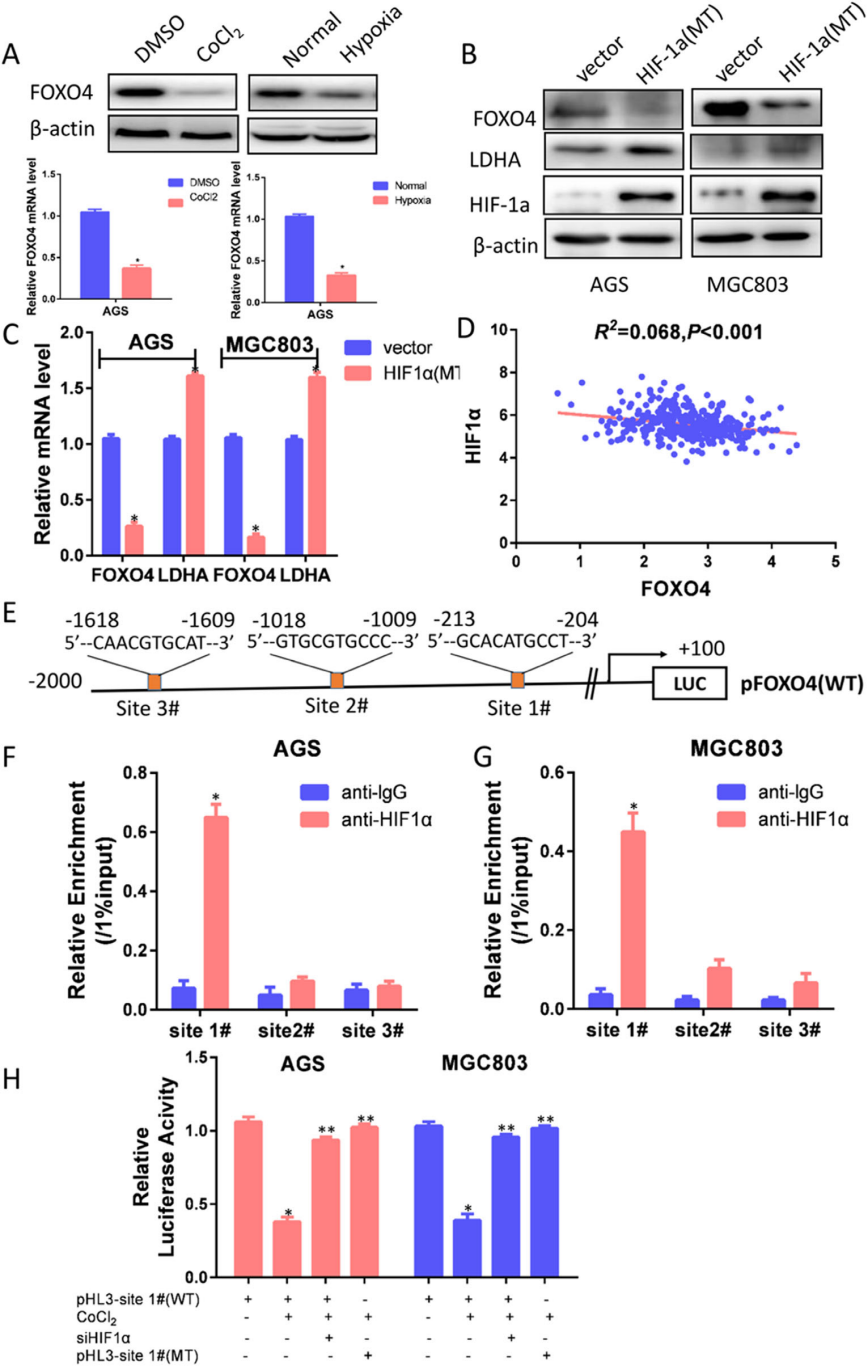
FOXO4 was directly regulated by the transcription factor HIF1α. (A) FOXO4 was significantly decreased under hypoxic cell culture induced by CoCl2 or 1% O2. (B, C) After transfection with a mutated HIF‐1α lacking the oxygen‐dependent degradation (ODD) domain, western blotting (B) and RT‐PCR (C) analysis indicated that mutated HIF‐1α decreased FOXO4 expression at both protein and transcriptional levels. (D)HIF‐1α expression was negatively correlated with FOXO4 expression in the TCGA database (*P <* *0.001*). (E) Three HIF‐1α transcription factor binding elements were identified in FOXO4 promoter by using the JASPAR CORE database. (F, G) ChIP assays revealed that HIF‐1α mainly bound to the first HRE site 5′‐GCACATGCCT‐3′ located from ‐204 to ‐213 bp. (H) Luciferase analysis indicated that FOXO4 promoter activity was decreased in cells treated with CoCl2 and was restored in cells silencing HIF‐1α expression. Consistent with these results, when we mutated 5′‐GCACATGCCT‐3′ sequence in FOXO4 promoter to 5′‐ATGTGCTAAC‐3′, the mutated promoter completely abolished the CoCl2 responsiveness of the construct. *WT*: Wild type FOXO4 promote. *MT*: Mutated FOXO4 promote. *P < 0.05.**P > 0.05

Hypoxia is a hallmark of solid tumors and promotes tumor progression and metastasis of various cancers.^14^ To understand the transcriptional regulation of FOXO4 expression in GC, we treated GC cells with 100μmol CoCl2, which significantly decreased FOXO4 expression at both protein and mRNA levels. Consistently, FOXO4 expression was significantly decreased in hypoxic cell culture (Figure 6A). We transfected a mutated HIF‐1α that lacked the oxygen‐dependent degradation (ODD) domain by mutation proline 564 to alanine, proline 402 to alanine, and it was not degraded under normoxia. The mutated HIF‐1α decreased FOXO4 expression at both transcriptional and protein levels (Figure 6B, C). Moreover, HIF‐1α expression was inversely associated with FOXO4 expression status in the TCGA database (*P <* *0.001*) (Figure 6D). To understand whether FOXO4 is a direct target of HIF‐1α, we analyzed the FOXO4 promoter sequence using the JASPAR CORE database. Three HIF‐1α transcription factor binding sites were found (Figure 6E). ChIP assays revealed that HIF‐1α mainly bound to the first HRE site 5′‐GCACATGCCT‐3′ located from ‐204 to ‐213 bp (Figure 6F,G). We then cloned the FOXO4 promoter sequence from +100 to ‐500 bp into the pGL‐3 luciferase reporter construct. Luciferase analysis indicated that FOXO4 promoter activity was decreased in cells treated with CoCl2 and was restored in cells silencing HIF‐1α expression. Consistent with these results, when we mutated the 5′‐GCACATGCCT‐3′ sequence in FOXO4 promoter to 5′‐ATGTGCTAAC‐3′, the mutated promoter abolished the inhibitory effect of CoCl2 on the construct (Figure 6H). This indicated that the 5′‐GCACATGCCT‐3′ site within the FOXO4 promoter mediated the HIF‐1α response.

## 4 DISCUSSION

The survival of GC is still dismal. More than half of patients with GC will develop metastasis after radical gastrectomy, which is the main cause of death for patients with GC. Studies of prognostic factors for GC patients and of predictors for risk of recurrence and metastasis are urgently needed. Our previous research has demonstrated that FOXO4 may be as a novel predictor for patients with GC.^6^ In the present study, we confirmed FOXO4 protein level in GC by immunohistochemistry. FOXO4 expression was significantly decreased in GC, and loss of FOXO4 expression correlated with lymph node metastases and larger tumor size. More importantly, FOXO4 status was confirmed as an independent prognostic biomarker in GC.

Aerobic glycolysis has been highlighted as a hallmark of cancer cells in recent decades.^15^ Aerobic glycolysis provides adequate ATP for cancer cell proliferation and formed an acid microenvironment to facilitate metastasis. Aberrant expression of some genes may be involved in glycolysis regulation and confer a growth advantage for cancer cells.^16^ As FOXO4 expression is closely associated with tumor diameter and lymph node metastases, we speculate that loss of FOXO4 expression may enhance glycolysis activities in GC and provide the following evidence. First, restored FOXO4 expression significantly decreased glycolysis rate as demonstrated by impaired glucose uptake, lactate, and ATP production in GC cells. These results were further confirmed by Seahorse analysis that showed that FOXO4 expression enhanced mitochondrial respiration and impaired glycolysis. Second, glycolysis is considered to be the theoretical basis for the most accurate imaging technique for tumor assessment in clinical practice, namely PET of 18F fluorodeoxyglucose (18F‐FDG) uptake.^17^ Using *in vivo* mice model and patients’ sample study indicated that FOXO4 status was negatively associated with SUVmax value. Third, we found that the expression of FOXO4 transcriptionally regulated the glycolytic enzyme LDHA. LDHA mainly catalyzes pyruvate to lactate, which is the key rate‐limiting enzyme in glycolysis pathway.^18^

Hypoxia is a common microenvironmental factor in solid tumors and is also found in GCs.^19^, ^20^ Accumulating evidence demonstrates that hypoxia can trigger HIF‐1α and its downstream targets to increase blood vessel formation, aggressiveness, and resistance to treatment.^21^ Hypoxia also induces glycolysis in some cancer cells, further increasing their survival advantage.^22^ To establish whether FOXO4 is a response to hypoxia, we treated GC cells with 1% O2 or CoCl2 and found that FOXO4 expression was significantly downregulated in hypoxia. The central feature in response to hypoxia is the induction of transcription factor HIF‐1α. We transfected GC cells with mutated HIF‐1α, and found that HIF‐1α decreased FOXO4 expression at both transcriptional and protein levels. Moreover, FOXO4 expression was inversely associated with HIF‐1α expression in the TCGA database. To confirm the transcriptional regulation of FOXO4 expression by HIF‐1α in GC, we analyzed HIF‐1α binding sites on FOXO4 promoter. We found three HIF‐1α putative binding elements and confirmed the first site as the HIF‐1α binding site in FOXO4 promoter. In general, HIF‐1α promotes transcription of target genes by recruiting p300 HAT. However, there have some researches about HIF‐1α transcriptional regulates tumor suppressor. For example, the adenomatous polyposis coli (APC) is a classic tumor suppressor, and it was repressed by HIF‐1α via a functional HRE on the its promoter in osteosarcoma and colon cancer cells.^23^ RECK is a tumor suppressor and hypoxia significantly downregulates RECK mRNA and protein expression, while the effect is abolished by knockdown HIF‐1α with respective siRNAs. Mechanistically,HIF‐1α binds directly to the HRE site of RECK promoter.^24^ Also, HIF‐1α impacts NF‐κB‐dependent gene status to regulate innate immunity signals.^25^ Tang *et al*. reported that HIF‐1α was a downstream target of FOXO4,^26^ while we provided sufficient evidence that FOXO4 is a transcriptional target of HIF‐1α. We speculated that HIF‐1α and FOXO4 may form a loop to stimulate GC progression.

## 5 CONCLUSION

We have provided a critical evidence of the FOXO4 in GC progression, and shown that the HIF‐1α‐FOXO4‐LDHA axis plays an critical role by promoting aerobic glycolysis. Therefore, this novel signaling pathway may be as a novel biomarker and therapeutic target for GC.

## AUTHOR CONTRIBUTIONS

Literature search and study design: XHW and ZHJ. Data analysis: XHW, ZHJ, YZ, HMY. Data collection: HMY, LHX, XHW. Experimental study: XHW, ZHJ, YZ, HMY, YZ. Manuscript writing: YHW, ZHJ. All authors read and approved the final manuscript.

## FUNDING

This work was supported by grants from Jiangsu Province Science and Education Health Project (Grant No. QNRC2016468) and Yancheng Medical Science and Technology Development Project in 2018 (Grant No. YK2018006).

## AVAILABILITY OF DATA AND MATERIALS

The datasets generated and/or analyzed during the current study are available from the corresponding author on reasonable request.

## ETHICS APPROVAL AND CONSENT TO PARTICIPATE

The study protocol was approved by the Institutional Review Board of The First People's Hospital of Yancheng.

## CONSENT FOR PUBLICATION

Not applicable.

## CONFLICT OF INTEREST

The authors declare no conflict of interest

## Supporting information

Supporting Information

Supporting Information
