# Supplementary material for: Hypoxia‐induced FOXO4/LDHA axis modulates gastric cancer cell glycolysis and progression
Source: Clin Transl Med. 2021 Jan 15;11(1):e279. doi: 10.1002/ctm2.279 (PMC7809603; doi:10.1002/ctm2.279)
Supplement: Supplementary file 1 — Supporting Information [file CTM2-11-e279-s001.docx]

**Table S1. All Primers for Glycolytic RT-PCR analysis**

| **Primers** | **Sequences(5'to3')** |
| --- | --- |
| GLUT1F | CTTTGTGGCCTTCTTTGAAGT |
| GLUT1R | CCACACAGTTGCTCCACAT |
| HK2F | GATTGTCCGTAACATTCTCATCGA |
| HK2R | TGTCTTGAGCCGCTCTGAGAT |
| GLUT4F | TGGAAGGAAAAGGGCCATGCTG |
| GLUT4R | CAATGAGGAATCGTCCAAGGATG |
| G6PF | GGTACACAGGCAAGACCATC |
| G6PR | GTTTTGGCAATGTGAGTTCC |
| PFKMF | GATACTATGGATGATCCAGACAC |
| PFKMR | GACAGCAATGGCTTTGCCAATC |
| PFKLF | CACAGGTGCCAACATCTTCCGCA |
| PFKLR | TCATGTCGGTGCCGCAGAAGTCG |
| PFKPF | AGAGGACCTTCGTTCTGGAGGT |
| PFKPR | GGGCACGGTTCTCCGAGAGTTT |
| ALDOAF | GTTATCAAATCCAAGGGCGGTGTT |
| ALDOAR | AGTCAGCTCCGTCCTTCTTGTAC |
| ALDOBF | CACCATTCAAGGGCTTGATGGCCT |
| ALDOBR | TTCCTGGATAGCGAGGCTGGAT |
| GAPDHF | GCACCGTCAAGGCTGAGAAC |
| GAPDHR | TGGTGAAGACGCCAGTGGA |
| PGK1F | CAAGGTTAAAGCCGAGCCAGCCAA |
| PGK1R | GCCTTCTGTGGCAGATTGACTCC |
| PGAM1F | ATGATGTCCCACCACCTCCGAT |
| PGAM1R | ATCCTTCAGACTCTCACAGGAG |
| ENO1F | GCTCCGGGACAATGATAAGACTCG |
| ENO1R | CTGTTCCATCCATCTCGATCATC |
| ENO2F | TGAAGGCAGTGGACCACATCAACT |
| ENO2R | AGAGACACACCCAGGATGGCATT |
| PKM2F | CAAAGGACCTCAGCAGCCATGTC |
| PKM2R | GGGAAGCTGGGCCAATGGTACAGA |
| LDHAF | TGGAGATTCCAGTGTGCCTGTATGG |
| LDHAR | CACCTCATAAGCACTCTCAACCACC |
| LDHBF | GGAAGGAAGTGCATAAGATGGTGG |
| LDHBR | CCCCTTTACCATTGTTGACACG |
| PKM1F | CTATCCTCTGGAGGCTGTGC |
| PKM1R | CCATGAGGTCTGTGGAGTGA |
| FOXO4F | AGTCTGAGGTGCTGGCGGAG |
| FOXO4R | GGTGGTGGCGTATCAGAGGTG |
| actinF | CTACGTCGCCCTGGACTTCGAGC |
| actinR | GATGGAGCCGCCGATCCACACGG |
