# Supplementary figures and images for: Hypoxia‐induced FOXO4/LDHA axis modulates gastric cancer cell glycolysis and progression
Source: Clin Transl Med. 2021 Jan 15;11(1):e279. doi: 10.1002/ctm2.279 (PMC7809603; doi:10.1002/ctm2.279)

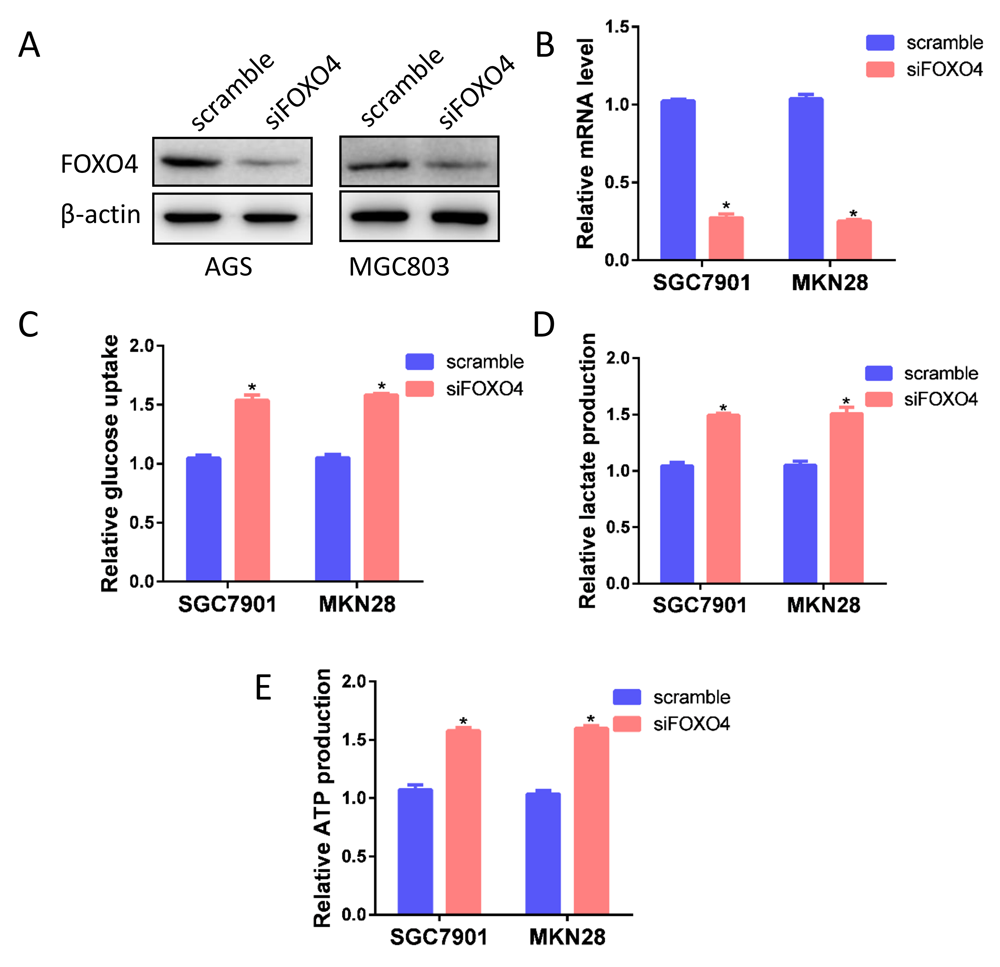

Supplement: Supplementary file 2 — Supporting Information [file CTM2-11-e279-s002.tif]
